# Air pollution and adverse cardiac remodeling: clinical effects and basic mechanisms

**DOI:** 10.3389/fphys.2015.00162

**Published:** 2015-05-20

**Authors:** Yonggang Liu, Jamie M. Goodson, Bo Zhang, Michael T. Chin

**Affiliations:** ^1^Division of Cardiology, Department of Medicine, University of WashingtonSeattle, WA, USA; ^2^Department of Pathology, University of WashingtonSeattle, WA, USA

**Keywords:** air pollution, diesel exhaust particulates, cardiac remodeling, cardiac hypertrophy, heart failure

## Abstract

Exposure to air pollution has long been known to trigger cardiovascular events, primarily through activation of local and systemic inflammatory pathways that affect the vasculature. Detrimental effects of air pollution exposure on heart failure and cardiac remodeling have also been described in human populations. Recent studies in both human subjects and animal models have provided insights into the basic physiological, cellular and molecular mechanisms that play a role in adverse cardiac remodeling. This review will give a brief overview of the relationship between air pollution and cardiovascular disease, describe the clinical effects of air pollution exposure on cardiac remodeling, describe the basic mechanisms that affect remodeling as described in human and animal systems and will discuss future areas of investigation.

## Introduction

Exposure to various forms of air pollution has been linked to cardiovascular disease in multiple human and animal studies (reviewed in Chin, 2015). Air pollution consists of many gaseous and particulate components and can vary significantly in chemical composition depending on local environmental conditions. Particulate matter air pollution, comprised primarily of fine particulates derived from combustion, is a major component of ambient air pollution and exhibits the most cardiotoxic effects (Brook et al., 2004, 2010; Miller et al., 2007; Chin, 2015). Ambient particulates are generally categorized on the basis of size, and most toxicity studies have focused on particulates less than or equal to 10 μm in diameter, referred to as PM_10_ (reviewed in (Brook et al., 2004)), or a subset of PM_10_, particulates less than or equal to 2.5 μm in diameter, referred to as PM_2.5_. Particles in this size range are especially toxic because of their ability to reach the alveoli, where they activate multiple pathophysiological mechanisms (Brook et al., 2010; Chin, 2015). Both acute and chronic PM_2.5_ exposure have been associated with exacerbation of ischemic heart disease (Xie et al., 2015), heart failure (Shah et al., 2013), cerebrovascular disease (Stafoggia et al., 2014), thrombosis (Baccarelli et al., 2008), hypertension (Bellavia et al., 2013), and arrhythmias (Bartell et al., 2013). This review will focus specifically on physiological mechanisms that are activated by PM exposure to promote adverse cardiac remodeling (Atkinson et al., 2013).

The basic physiological mechanisms by which PM_2.5_ promotes cardiovascular disease have been studied extensively in both humans and animal models, though many questions remain (Brook et al., 2010; Chin, 2015). The most commonly accepted mechanism is that inhalation into the lungs promotes a local inflammatory response that then “spills over” into the circulation, where soluble and cellular mediators can then promote systemic vascular oxidative stress and inflammation that affect the heart and vasculature. This systemic effect can also be amplified by effects on adipose and liver tissue, promoting the release of adipokines and acute phase reactants, which can alter vascular tone, resulting in insulin resistance, dyslipidemia and hypercoagulability (reviewed in Brook et al., 2010). Another mechanism involves activation of autonomic reflex arcs in the lung, mediated by TRP receptors, which results in elevated sympathetic nervous system activity and reduced parasympathetic nervous system activity that then promotes vasoconstriction, endothelial dysfunction, platelet aggregation, hypertension and arrhythmogenesis. A third mechanism involves exit of ultrafine particulates or soluble components associated with these particulates from the lungs into the circulation where they directly promote vasoconstriction, endothelial dysfunction, vascular oxidative stress, hypertension and possibly atherosclerosis and platelet aggregation. A fourth mechanism involves the epigenetic modification of DNA in circulating cells and target tissues, resulting in altered gene expression patterns that promote conditions such as hypertension (Bellavia et al., 2013). These mechanisms and their downstream effects on cardiac remodeling are shown in Table 1. The specific mechanisms by which air pollution exposure affects cardiac remodeling can be indirect and in conjunction with other processes such as ischemia or hypertension or direct and mediated by systemic inflammatory mediators and/or epigenetic reprogramming.

**Table 1:** **Pathophysiological effects of particulate matter air pollution and their relevance to cardiac remodeling**.

**Pathophysiological mechanisms**	**Downstream effects relevant to cardiac remodeling**
Local pulmonary inflammatory response with systemic spread (Mutlu et al., 2007; Urch et al., 2010; Kampfrath et al., 2011; Tsai et al., 2012)	- ↑Systemic inflammation - Vascular oxidative stress - Release of acute phase reactants - ↑Coagulation - Insulin resistance
Activation of autonomic reflex arcs in lungs (Rhoden et al., 2005; Ghelfi et al., 2008; Zhong et al., 2015)	- ↑ Sympathetic nervous system activity - ↓ Parasympathetic nervous system activity - ↑ Vasoconstriction - Endothelial dysfunction - Platelet aggregation - Hypertension - Arrhythmogenesis
Release of ultrafine particles or particulate components into the bloodstream (Nemmar et al., 2002, 2003a,b)	- ↑Systemic inflammation - Vascular oxidative stress - Endothelial dysfunction - Hypertension - Platelet aggregation - Atherosclerosis (?)
Exposure triggers changes in epigenetic regulation (Yauk et al., 2008; Bellavia et al., 2013; Zhong et al., 2015)	- Changes in leukocyte gene expression, affecting immune response

## Epidemiological associations between particulate matter exposure and abnormal cardiac remodeling

In one study, the relationship between traffic-related air pollution exposure, cardiac hypertrophy and left ventricular function was specifically analyzed, using an estimation of exposure based on proximity to major roadways and magnetic resonance imaging in a multi-ethnic patient population known to be free of coronary artery disease. Participants living within 50 m of a major roadway were found to have increased left ventricular mass index but no change in ejection fraction (Van Hee et al., 2009). A follow up study identified associations between single nucleotide polymorphisms in the genes encoding the type I angiotensin II receptor (AGTR1) and arachidonate 15-lipoxygenase (ALOX15) and increased left ventricular mass associated with living in proximity to major roadways (Van Hee et al., 2010). These genes are important in vascular function, inflammation and oxidative stress, implying that the observed increase in LV mass may arise through these mechanisms. Interestingly, exposure to PM_10_ but not PM_2.5_ is associated with development of heart failure in an English cohort (Atkinson et al., 2013).

## Immunological effects of PM exposure

Systemic inflammation is a common mechanism that causes both direct and indirect effects on cardiac remodeling (Table 1). Exposure can trigger both the adaptive and innate immune responses. In mice, PM_2.5_ exposure releases oxidized phospholipids in the lungs that then activate Toll-like Receptor 4 (TLR4)/NADPH oxidase-dependent mechanisms to promote systemic inflammation (Kampfrath et al., 2011). In primary mouse macrophages from Toll-like Receptor 2 (TLR2) knockout mice, the Tumor Necrosis Factor-α (TNF-α) and Interleukin-6 (IL-6) response to PM_2.5_ exposure is blunted (Shoenfelt et al., 2009). Upregulation of IL-6 in mice in response to PM_10_ intratracheal instillation has been associated with an increase in coagulation (Mutlu et al., 2007), demonstrating how inflammation may provoke vascular occlusion and ischemia that can influence cardiac remodeling.

A study examining the response of transformed human bronchial epithelial cells to diesel exhaust particulate exposure showed an induction of CYP1A1 and downstream increased expression of IL-6 and IL-8 (Totlandsdal et al., 2010). In humans, short term PM_10_ exposure is associated with increased levels of circulating IL-1β, IL-6 and TNF-α (Tsai et al., 2012). Controlled short-term exposure of human subjects to PM_2.5_ also leads to elevations in circulating IL-6 (Urch et al., 2010). Transcriptional profiling of peripheral blood mononuclear cells from human subjects exposed to PM_2.5_ reveals changes in expression of genes associated with inflammation, oxidative stress and coagulation (Peretz et al., 2007; Pettit et al., 2012), suggesting that these mechanisms are clinically relevant. Chronic human exposure to PM_2.5_ leads to hypermethylation of the TLR2 promoter in leukocytes and promotes autonomic dysfunction (Zhong et al., 2015). Other human studies have not shown changes in circulating inflammatory mediators, however, indicating that these studies are not always consistent (Mills et al., 2008). The reason for these variable inflammatory reactions may be context dependent, as the time of exposure and chemical composition of PM used in the studies may alter the response.

## Particulate matter air pollution, ischemic injury and adverse cardiac remodeling

Ischemic injury to myocardium is well known to lead to adverse cardiac remodeling. Occlusion of coronary artery blood flow to myocardium leads to infarction and myocyte necrosis, followed by inflammation, scarring and remodeling of the heart to compensate for reduced contractile function. Occlusion can occur as the result of progressive atherosclerosis, acute vascular thrombosis, or a combination of the two, which is most likely. Endothelial dysfunction resulting in vasoconstriction can also play a role. PM_2.5_ exposure has been shown to exacerbate ischemic injury to myocardium in many studies, through effects on atherosclerosis, thrombosis and vasoconstriction (Brook et al., 2010; Chin, 2015).

An early study demonstrated that exposure of dogs to concentrated ambient particles leads to increased ST segment elevation after 5 min coronary artery occlusion (Wellenius et al., 2003). The combination of a high fat diet and chronic PM_2.5_ exposure has been shown to increase vascular oxidative stress and promote progression of atherosclerosis (Sun et al., 2005). Ultrafine particles (PM_0.1_) have been shown to promote oxidative stress and early atherosclerosis (Araujo et al., 2008). Studies on experimental atherosclerosis in two different mouse models have shown that long term exposure to PM_2.5_ in the form of inhaled concentrated ambient particulates (CAPs) promotes increased CD36 expression in plaque macrophages that facilitates greater uptake and accumulation of an oxidized form of cholesterol in atherosclerotic lesions (Rao et al., 2014). Chronic exposure to CAPs can also promote inflammatory monocyte egress from bone marrow, production of reactive oxygen species and subsequent vascular dysfunction as measured by isolated aortic ring contraction through TLR4 activation of NADPH oxidase in mice (Kampfrath et al., 2011).

Acute ischemic events that lead to myocardial infarction and adverse cardiac remodeling in patients are generally mediated by atherosclerotic plaque rupture and occluding thrombus formation on the surface of the ruptured plaque. In a hamster model, intratracheal administration of diesel exhaust particulates (DEPs) has been shown to enhance experimental thrombus formation (Nemmar et al., 2003a). Follow up studies indicated that the enhanced thrombosis is mediated by production of histamine in the lungs (Nemmar et al., 2003b) and that stabilization of mast cells reduces this effect (Nemmar et al., 2004). In mice, intratracheal administration of PM_10_ has been shown to increase lung production of IL-6, reduce bleeding time, prothrombin time and activated partial thromboplastin time, increase plasma fibrinogen and increase activities of coagulation factors II, VIII and X (Mutlu et al., 2007). In hamsters, PM_2.5_-induced thrombosis is also reportedly further enhanced by angiotensin II-induced hypertension (Nemmar et al., 2011). In mice, either inhalation or intratracheal instillation of CAPs leads to increases in lung and adipose tissue PAI-1, implying that reduction in fibrinolysis may also play a role (Budinger et al., 2011). A more recent study in mice showed that inhaled CAPs promotes activation of the sympathetic nervous system and systemic catecholamine release, which in turn activates β2-adrenergic receptor-dependent release of IL-6 from alveolar macrophages to promote a prothrombotic state as measured by FeCl_3_-induced carotid artery time to loss of blood flow (Chiarella et al., 2014). Acute exposure of healthy human volunteers to DEPs promotes an increase in thrombus formation, platelet-neutrophil and platelet-monocyte aggregates as measured *ex vivo* in a Badimon chamber, implying an increase in platelet activation (Lucking et al., 2008).

## Particulate matter air pollution, hypertension and adverse cardiac remodeling

Hypertension is well known to promote adverse cardiac remodeling, by inducing concentric hypertrophy, eccentric hypertrophy, systolic and diastolic dysfunction (Santos and Shah, 2014). Reported effects of PM exposure on blood pressure vary, which may reflect differences in experimental methodology but may also reflect a need for interaction with other factors (Brook et al., 2010). Short-term (10 weeks) exposure to CAPs reportedly promotes vascular oxidative stress, increases hypertension in response to angiotensin II and activates Rho/ROCK in rats (Sun et al., 2008). Acute exposure of rats to CAPs (4 days) increases expression of endothelin receptor-A in rat hearts, which correlates with an increase in blood pressure, implying a role for endothelin in PM_2.5_-mediated hypertension (Ito et al., 2008). Exposure of spontaneously hypertensive rats to ultrafine particles for 24 h leads to increases in blood pressure 1–3 days after exposure that are associated with increased expression of endothelin-1 messenger RNA in lungs and increased circulating levels of plasma renin and angiotensins I and II (Upadhyay et al., 2008). Short-term exposure (12 weeks) of mice to CAPs confirms the potentiating effect of PM_2.5_ exposure on angiotensin II-induced hypertension in rats, through a similar Rho/ROCK mechanism, but also promotes increased cardiac hypertrophy and collagen deposition (Ying et al., 2009). Long-term (6 months) exposure to CAPs leads to activation of the sympathetic nervous system, hypothalamic inflammation and systemic hypertension in mice (Ying et al., 2014). The source of air pollution is likely to affect toxicity, however, as some experiments using DEPs have not shown any effect of either acute (7 days), short term (1 month) or chronic exposure (6 months) on cardiac remodeling in mice in both an angiotensin II infusion model and a transverse aortic constriction (TAC) model. This discrepancy between DEPs and CAPs likely reflects additional toxins that associate with CAPs in the atmosphere that are not initially associated with DEPs (Liu et al., 2013). Controlled exposure studies in human subjects have shown that acute exposure to CAPs in conjunction with ozone leads to transient diastolic hypertension, possibly due to autonomic imbalance (Urch et al., 2005) and that short-term, controlled human exposure to PM_2.5_ CAPs resulted in an increase in systolic blood pressure that correlates with reduced DNA methylation in Alu repetitive elements in circulating leukocytes (Bellavia et al., 2013).

## Diffuse myocardial damage, cardiac fibrosis and abnormal cardiac remodeling

Abnormal cardiac remodeling due to cardiac fibrosis can develop in response to myocardial injury, oxidative stress, mechanical stress and/or the influence of circulating mediators and is associated with changes in DNA methylation (reviewed in Tao et al., 2014). A hallmark of this process is activation of cardiac fibroblasts to generate excessive amounts of extracellular matrix proteins. Cardiac fibrosis can then lead to both systolic and diastolic dysfunction. In Wistar–Kyoto rats, protracted, repeated inhalation exposure to oil combustion-derived particulate matter leads to the development of multifocal, inflammatory, degenerative and fibrotic lesions in the myocardium (Kodavanti et al., 2003). A follow up study indicated that PM-associated zinc appears to be an important contributor to focal subepicardial inflammation, myocardial degeneration, fibrosis and mitochondrial DNA damage after 16 weeks of intratracheal instillation. There were also measurable effects on mRNA expression for genes involved in signaling, ion channel function, oxidative stress, mitochondrial fatty acid metabolism and cell cycle regulation (Kodavanti et al., 2008). A 3-month exposure of mice to CAPs has been shown to exacerbate angiotensin II-induced cardiac hypertrophy and fibrosis in a Rho kinase dependent manner (Ying et al., 2009). A 9-month exposure of mice to CAPs results in increased ventricular size along with systolic and diastolic dysfunction at the organ level, attributable to myocardial fibrosis at the tissue level and associated with decreased antioxidant activity as well as reduced contractile reserve. At the cellular level, isolated myocytes showed reduced fractional shortening, decreased shortening velocity and increased relaxation time. At the molecular level, expression of hypertrophic and profibrotic markers were increased (Wold et al., 2012). Exposure of rats to diluted motorcycle exhaust leads to increased heart weight, wall thickness and histological evidence for focal cardiac degeneration and necrosis, mononuclear cell infiltration and fibrosis. Cardiac antioxidant enzyme activities for glutathione S-transferase, superoxidase dismutase and glutathione peroxidase are also reduced. Analysis of mRNA expression in these hearts reveals increased levels of interleukin-β, atrial natriuretic peptide, collagen type I, collagen type III, connective tissue growth factor and transforming growth factor β1 transcripts, suggesting that exposure to motorcycle exhaust promotes hypertrophy and fibrosis through mechanisms involving oxidative stress and inflammation (Chen et al., 2013), although transcript levels do not necessarily reflect amount of protein expressed.

## Abnormal cardiac remodeling in adults after early life diesel exhaust exposure

Early life exposure to air pollution is known to cause low birth weight (Dadvand et al., 2013) and abnormal lung development in human populations (Gauderman et al., 2004, 2007). A recent study from the Netherlands found that children that are chronically exposed to PM_2.5_ have increased diastolic blood pressure (Bilenko et al., 2015), and a Boston newborn study found that third trimester maternal exposure to PM_2.5_ is correlated with increased newborn systolic blood pressure (van Rossem et al., 2015). A study of mouse exposure to DEPs during several discrete developmental windows demonstrated an increased susceptibility to pressure overload-induced heart failure in mice that were exposed *in utero* and up to the age of weaning. There was no change in basal cardiac function prior to TAC surgery. The magnitude of the effect was similar to that seen in animals exposed throughout life from conception through adulthood. Exposure during the adult period from weaning through the age of 12 weeks had no effect on susceptibility to heart failure. The hearts of animals exposed early in life showed increased heart weight to body weight ratios and interstitial fibrosis but no significant change in myocyte cross sectional area when compared to those from control mice exposed to filtered air. Interestingly, the lungs of mice exposed early in life showed differential induction of interleukin-6 expression after TAC surgery (Weldy et al., 2013). A follow up study in which exposure to DEPs was limited solely to gestation demonstrated that *in utero* exposure alone is sufficient to convey a long lasting susceptibility to myocardial fibrosis and heart failure when *in utero*-exposed adult mice were subjected to TAC surgery, suggesting that mediators in the maternal circulation could cross the placenta and promote long lasting susceptibility in the developing embryo hearts. Further findings include a baseline increased body weight to tibia length ratio and paradoxically lower baseline blood pressure in adult male animals. Histological examination of hearts from exposed animals showed increased interstitial fibrosis but no significant difference in myocyte cross sectional area. Examination of placental and fetal tissue from exposed dams showed a higher rate of fetal reabsorption, a lower average placental weight, increased incidence of placental hemorrhage, an increase in leukocyte infiltration and an increase in vascular oxidative stress (Weldy et al., 2014).

## Future challenges in understanding the role of air pollution in adverse cardiac remodeling

**Figure 1:**
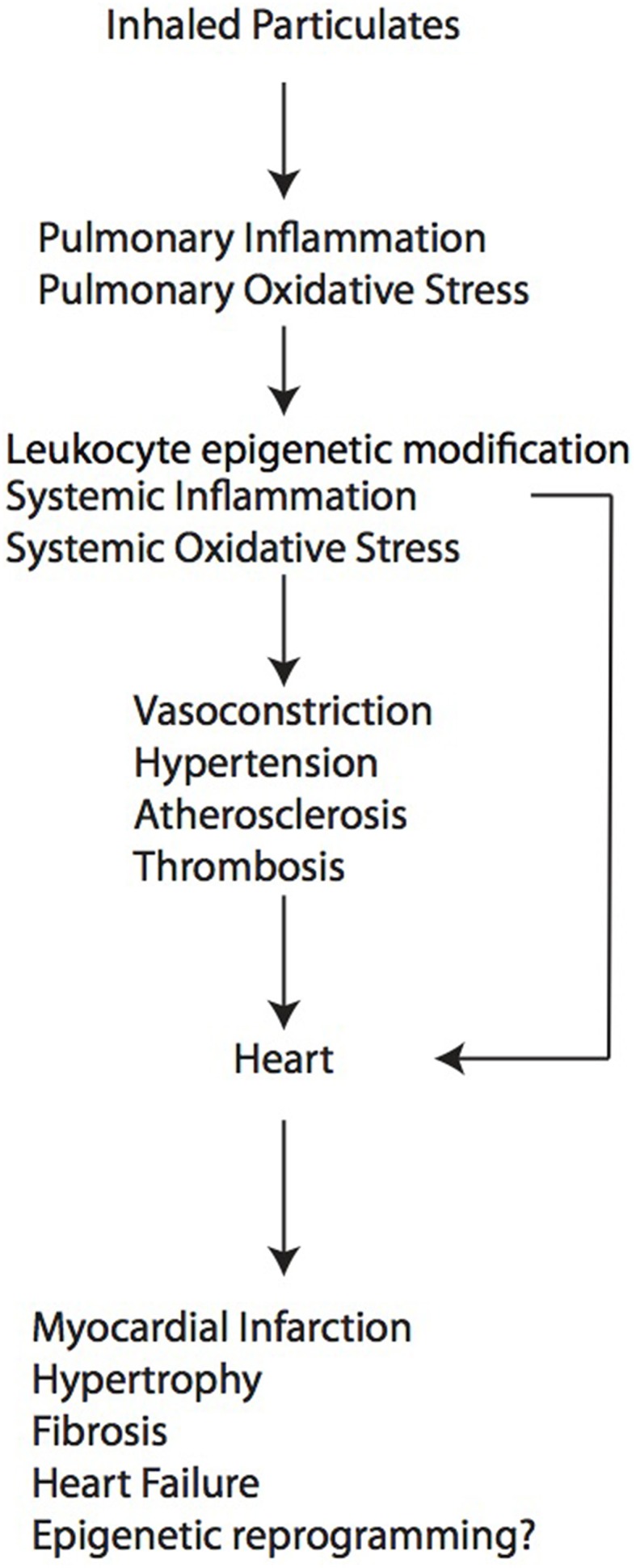
**A working model of how air pollution exposure promotes adverse cardiac remodeling**.

As described above, particulate air pollution can cause adverse cardiac remodeling through multiple indirect mechanisms, by exacerbating processes known to promote injury to myocardium such as atherosclerosis, vasoconstriction, thrombosis and hypertension (Figure 1). At present, however, little is known about effects on cardiomyocytes and cardiac fibroblasts at the cellular and molecular level, especially during development. For example, it is not known whether the cardiac fibrosis that develops in some models is due to a primary activation of cardiac fibroblasts or whether it reflects a primary injury to cardiomyocytes, resulting in cell death and replacement fibrosis. In the case of early life exposure, the molecular events that occur in the developing heart that confer later susceptibility to heart failure induced by pressure overload in the adulthood are completely unknown. Since air pollution exposure is also known to cause epigenetic modification in circulating leukocytes (Tarantini et al., 2009; Bellavia et al., 2013) and sperm (Yauk et al., 2008), and dynamic DNA methylation changes orchestrate cardiomyocyte development, maturation and disease (Gilsbach et al., 2014), future studies directed toward epigenetic regulation in cardiomyocytes may elucidate further the pathways and events that are responsible for pathological cardiac remodeling.

### Conflict of interest statement

The authors declare that the research was conducted in the absence of any commercial or financial relationships that could be construed as a potential conflict of interest.
